# Impact of Delirium on Pediatric Critical Care Outcomes and Management Strategies: A Systematic Review and Meta‐Analysis

**DOI:** 10.1155/jonm/9915593

**Published:** 2026-05-25

**Authors:** Cheng Yang, Jiawei Li, Qin Zeng

**Affiliations:** ^1^ Department of Pediatric Intensive Care Unit Nursing, West China Second University Hospital, Sichuan University, Chengdu, China, scu.edu.cn; ^2^ Key Laboratory of Birth Defects and Related Diseases of Women and Children (Sichuan University), Ministry of Education, Chengdu, China, meb.gov.tr; ^3^ Department of Pediatric Outpatient Nursing, West China Second University Hospital, Sichuan University, Chengdu, China, scu.edu.cn; ^4^ Department of Pediatric Gastroenterology Nursing, West China Second University Hospital, Sichuan University, Chengdu, China, scu.edu.cn

**Keywords:** clinical outcomes, critical care nursing, delirium, early detection, nursing management, patient safety, pediatric intensive care, quality of life

## Abstract

**Background:**

Delirium in the pediatric intensive care units (PICUs) poses substantial challenges for nursing care and has a profound impact on key clinical metrics, including the period of assisted breathing support, total hospitalization time, and child survival rates. Effective nursing strategies are essential to improve patient prognosis and quality of life.

**Aim:**

We perform a systematic review and meta‐analysis to examine how delirium relates to clinical outcomes in PICU patients and to map out the corresponding nursing management strategies.

**Design:**

A systematic review and meta‐analysis is conducted following the Preferred Reporting Items for Systematic Reviews and Meta‐Analyses (PRISMA) 2020 guidelines.

**Methods:**

Up to January 18, 2024, a comprehensive search of Cochrane Library, PubMed, Embase, and Cumulative Index to Nursing and Allied Health Literature (CINAHL) was conducted following the population, exposure, comparison, outcomes, and study design (PECOS) structure. Data were extracted with a standardized form, study quality was appraised using the Newcastle–Ottawa Scale, and analyses were carried out in STATA 17.0. The entire process complied with the PRISMA 2020 checklist.

**Results:**

Among 14 studies (6019 children), delirium significantly increased mechanical ventilation risk (odds ratio [OR] = 5.68, 95% confidence interval [CI]: 4.20–7.69, *p* < 0.01), prolonged intensive care unit (ICU) stay (standardized mean difference [SMD] = 0.61, 95% CI: 0.53–0.70, *p* < 0.01), and raised mortality (OR = 5.09, 95% CI: 1.81–14.32, *p* < 0.01). Limited data precluded meta‐analysis of postdischarge quality of life. Neither the funnel plot nor Egger’s test revealed significant publication bias.

**Conclusions:**

Delirium is a key predictor of nursing‐sensitive outcomes in pediatric ICU patients. Early screening and systematic nursing management (e.g., Cornell Assessment of Pediatric Delirium [CAPD] monitoring and family‐centered rehabilitation) reduce risks and improve recovery and quality of life. Nurses are essential in delirium management by conducting regular assessments, optimizing the care environment, and facilitating teamwork across disciplines. Healthcare institutions should incorporate delirium management protocols into routine nursing workflows and standardize quality‐of‐life evaluations.

## 1. Introduction

Delirium is defined as an acute, fluctuating disturbance of brain function, marked by changes in consciousness, difficulties in attention, sensory, and perceptual alterations, as well as hallucinations [1]. In adult populations, numerous studies have established that delirium correlates with unfavorable clinical outcomes, including increased mortality, longer durations of mechanical ventilation, prolonged hospitalization, elevated healthcare expenses, and a greater risk of enduring cognitive deficits [2–4].

While much of the existing literature focuses on adult populations, delirium can occur at any age, including in children, and its incidence is notably higher among pediatric patients. For instance, one study reported that the incidence of delirium in children hospitalized in the intensive care unit (ICU) for at least 48 h was as high as 69% [5]. This highlights the urgent need for enhanced clinical awareness and tailored management strategies specific to pediatric patients.

Children are a special and vulnerable population with ongoing physical and neurodevelopmental processes. The clinical features of delirium in children differ significantly from those in adults, often presenting with symptoms such as irritability, anxiety, lethargy, unresponsiveness, and agitation, which may be influenced by various physiological imbalances such as dopaminergic excess, abnormal cortisol secretion, and heightened stress responses [6]. Recent evidence suggests that specific delirium symptoms like lethargy, delayed response, and disorientation may serve as indicators of severity and critical illness in pediatric patients [7].

Given these challenges, nurses play a crucial role in the early detection, prevention, and management of delirium; consistent application of validated tools—such as the Cornell Assessment of Pediatric Delirium (CAPD)—is central to effective screening [8]; additionally, environmental modifications (e.g., sleep promotion and minimizing noise)[9], family involvement [10], and multidisciplinary collaboration [11] are essential strategies. The development of standardized nursing protocols for delirium assessment and intervention can facilitate timely recognition and treatment, ultimately reducing morbidity [12].

Despite the growing recognition of pediatric delirium, the current evidence base remains limited, with many studies characterized by small sample sizes and methodological heterogeneity [13–16]. Establishing clear clinical pathways, conducting staff training programs, and integrating delirium management into routine nursing workflows are vital steps needed to enhance early intervention. Nurses, as frontline caregivers, are pivotal in implementing these strategies—they should receive targeted education to improve their skills in recognizing early signs of delirium and managing risk factors.

A systematic review combined with meta‐analysis enables the synthesis of current evidence, elucidates the link between delirium and patient outcomes, and helps determine optimal nursing care strategies. Enhancing nursing protocols and staff training not only improves early detection but also promotes a culture of proactive delirium management, ultimately reducing complications and improving long‐term outcomes. The ultimate goal is to empower nurses and healthcare teams to establish effective clinical pathways, ensuring timely intervention and comprehensive care for pediatric patients at risk of delirium.

## 2. Methods

The study was conducted in accordance with the Preferred Reporting Items for Systematic Reviews and Meta‐Analyses (PRISMA 2020) [17] and registered on INPALSY (registration number INPLASY202370011). Subgroup analyses by study location and patient age were prespecified to explore potential sources of clinical heterogeneity.

### 2.1. Study Design Rationale

This systematic review and meta‐analysis was designed to synthesize observational evidence on the association between delirium and clinical outcomes in pediatric ICU patients. Delirium is a naturally occurring clinical syndrome rather than an intervention that can be randomly assigned. Therefore, randomized controlled trials (RCTs) are not feasible for addressing this research question, as it would be unethical to deliberately induce delirium in children. Consequently, we restricted inclusion to prospective cohort studies and retrospective cohort studies that included a nondelirium comparator group. This design allows for the estimation of predictive effects of delirium on outcomes such as mechanical ventilation, mortality, and length of stay while minimizing the risk of selection bias inherent to cross‐sectional or case–control designs.

### 2.2. Search Strategy

From inception to 18 January 2024, an exhaustive search was performed across PubMed, Cochrane Library, Embase, and Cumulative Index to Nursing and Allied Health Literature (CINAHL). The strategy combined Boolean logic with free‐text keywords, and key subject headings including “Delirium”, “Subacute Delirium”, “Mixed Origin Delirium”, “Child”, “Adolescent”, “Pediatrics”, “Child, Preschool”, “Paediatr∗”, “Toddler∗” and “Infant∗”. The corresponding search strategies adapted for PubMed, Embase, CINAHL, and Cochrane Library are provided in Supporting material 1.

To supplement the electronic database search, we manually screened the reference lists of all included studies and relevant systematic reviews. We also searched the clinical trial registry ClinicalTrials.gov (https://clinicaltrials.gov/) and used Google Scholar to identify grey literature, including conference abstracts, dissertations, and unpublished or ongoing studies.

### 2.3. Study Selection

The inclusion and exclusion criteria were defined a priori based on the population, exposure, comparison, outcomes, and study design (PECOS) framework as follows:

• P (Population): children admitted to the ICU;
• E (Exposure): children identified with delirium in the ICU through a validated screening tool;
• C (Comparator): children without delirium;
• O (Outcomes): mortality, ICU length of stay, duration of mechanical ventilation, proportion requiring mechanical ventilation, and postdischarge quality of life;
• S (Study design): prospective or retrospective cohort studies.

When the same cohort appeared in multiple reports, only the most recent and largest study was retained. Exclusion criteria were (1) absence of relevant outcome data; (2) nonoriginal publications (e.g., conference abstracts, reviews, and case reports); (3) studies of low quality (Newcastle–Ottawa Scale [NOS] score ≤ 4); and (4) inability to extract necessary data.

The literature search was performed as described in Section 2.2. All identified records were imported into EndNote X9 for duplicate removal. After deduplication, two reviewers (CY and JL) independently screened the titles and abstracts against the eligibility criteria. The inter‐rater agreement was assessed using Cohen’s kappa coefficient (*κ* = 0.85, indicating almost perfect agreement). If either reviewer considered a study potentially eligible, the full text was retrieved. Subsequently, the full texts were independently examined by both reviewers to decide on final inclusion. Any disagreements were resolved by a third reviewer (QZ) who re‐examined the records and the eligibility criteria to reach consensus. The reasons for exclusion at the full‐text stage were documented.

### 2.4. Data Extraction

Two researchers independently extracted data from eligible studies and settled discrepancies through discussion; if agreement remained elusive, a third reviewer was consulted. The coded dataset encompassed first author, publication year, study design, country, sample size, sex, age, screening instrument, and outcomes of interest, together with *P* values, 95% confidence intervals (CIs), odds ratios (ORs), or means ± standard deviations. All data were organized and stored using Microsoft Excel.

### 2.5. Quality Appraisal

Two assessors independently appraised study quality with the NOS [18]; disagreements were settled by a third assessor. This scale evaluates cohort studies across three domains—selection, comparability, and outcome assessment—with total scores ranging from 0 to 9: high quality (8–9 points), moderate quality (5–6 points), and low quality (≤ 4 points) [19].

### 2.6. Data Analysis

All analyses were conducted with Stata 17.0. For dichotomous outcomes such as mortality and the need for mechanical ventilation, pooled ORs and 95% CIs were calculated to adjust for varying sample sizes. Continuous outcomes—including ICU length of stay, ventilation duration, and postdischarge quality of life—were summarized using standardized mean differences (SMDs) with 95% CIs. Heterogeneity was quantified by the I^2^ statistic and Q test; because of clinical and methodological diversity, a random‐effects model was employed. Robustness was examined through leave‐one‐out sensitivity analyses [20]. Funnel plots and Egger’s test were used to assess publication bias when heterogeneity was substantial [21–23], whereas Harbord’s test was applied under low heterogeneity [24]. Subgroup analyses were performed to explore differences in predictors according to study location and patient age.

## 3. Results

### 3.1. Study Process

**FIGURE 1:**
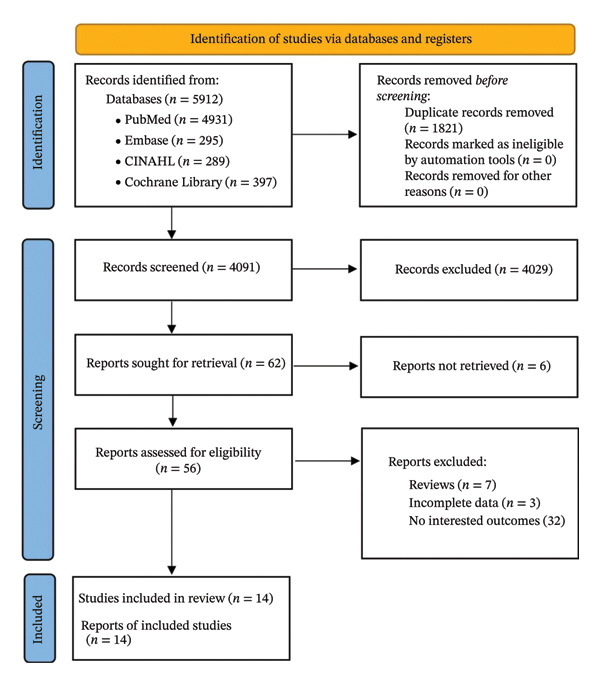
PRISMA 2020 flow diagram illustrating the study selection process (adapted from Page et al. [17]). Notes: The diagram details the number of records identified, screened, and excluded, and the final 14 studies included in the systematic review and meta‐analysis. Abbreviation: PRISMA, Preferred Reporting Items for Systematic Reviews and Meta‐Analyses.

After an initial yield of 5912 records, 1821 duplicates were removed. We excluded 4029 articles by reading titles and abstracts because of mismatches in research topics or content, and six articles were excluded because they were not retrieved. After careful reading, we additionally excluded 188 studies (7 reviews, 3 incomplete data, and 32 noninterested outcomes) and ultimately included 14 studies, encompassing a total of 5974 patients (Figure 1).

### 3.2. Study Profile

Fourteen cohort studies published within the past six years were incorporated into this review. The majority were conducted in the United States. Thirteen studies focused on children aged 18 years or younger, while one study included participants aged under 21 years. Sample sizes ranged from 43 to 1547 patients per study. Delirium was identified in 13 of these studies using the CAPD. Details of the delirium‐related outcomes assessed are presented in Table 1.

**TABLE 1:** General characteristics of the included studies.

Author	Publication year	Country	Type of study	Screening tool	Number of delirium cases (sample), *n*	Male, *n*	Age of sample (yr)	Outcome
Sudhakar et al. [25]	2022	India	Cohort study	p‐CAM‐ICU	12 (105)	72	11.10 ± 3.28	①②③
Silver et al. [26]	2020	USA	Cohort study	CAPD	56 (207)	121	13 (5–29)^∗^	④
Traube et al. [27]	2017	USA	Cohort study	CAPD	267 (1547)	880	0–2 38.3%	①②⑤
							2–5 20.5%	
							5–13 22.6%	
							> 13 18.6%	
Andrew et al. [28]	2022	USA	Cohort study	CAPD	634 (942)	529	0–2 61%	①②
							2–5 10%	
							5–13 15%	
							> 13 14%	
Dervan et al. [1]	2019	USA	Cohort study	CAPD	629 (908)	509	0–2 37%	①③⑤
							2–5 16%	
							5–13 20%	
							> 13 27%	
Patel et al. [29]	2017	USA	Cohort study	CAPD	95 (194)	114	0–2 41.8%	①
							2–5 24.7%	
							5–13 14.9%	
							> 13 18.6%	
Traube (2) et al. [30]	2017	USA	Cohort study	CAPD	209 (723)	537	0–2 48.7%	①③
							2–5 14.5%	
							5–13 19.9%	
							> 13 16.8%	
Traube (3) et al. [31]	2017	USA	Cohort study	CAPD	40 (319)	166	0–5 40.8%	②
							5–21 59.3%	
Christian et al. [32]	2022	USA	Cohort study	CAPD	29 (43)	18	< 1 51.2%	③
							1–2 32.5%	
							2–4 9.3%	
							5–9 2.3%	
							≥ 10 4.7%	
Siegel et al. [33]	2021	USA	Cohort study	CAPD	33 (147)	—	—	①
Alvarez et al. [34]	2018	USA	Cohort study	CAPD	56 (99)	52	< 1^∗^ 20.3%	①③⑤
							1^∗^‐1 24.2%	
							1–5 29.2%	
							6–12 13.2%	
							13–21 13.1%	
Huang et al. [35]	2023	China	Cohort study	CAPD	47 (109)	56	Delirium:	③④
							31.80 ± 10.60^∗^	
							No delirium:	
							36.50 ± 12.90^∗^	
Yontem et al. [36]	2021	Turkey	Cohort study	CAPD	14 (142)	85	43.00 (17.00–123.00)^∗^	③
Dervan et al. [37]	2022	USA	Cohort study	CAPD	235 (534)	293	3.30 (0.80–9.20)	①③④

*Note:* Age of sample: Results are shown as percentages (%), medians (IQRs), or means ± SDs.

Abbreviations: CAPD: Cornell Assessment for Pediatric Delirium; IQR: interquartile range; p‐CAM‐ICU: Pediatric Confusion Assessment Method for the Intensive Care Unit; SDs: standard deviations.

^∗^Months. Outcomes: ① mechanical ventilation rate; ② mortality; ③ length of ICU stay; ④ quality of life after discharge; and ⑤ duration of mechanical ventilation.

### 3.3. Quality Appraisal

All included studies were rated using the NOS, receiving scores ranging from 6 to 8 stars. Specifically, 2 studies were awarded 8 stars, and 8 studies were awarded 7 stars, both classified as high quality, while 4 studies received 6 stars, representing moderate quality. Table 2 illustrates the detailed quality assessment results.

**TABLE 2:** Quality of cohort studies according to the Newcastle–Ottawa Scale.

Author	Year	Selection	Intergroup comparability	Results of measurement	NOS total score
Sudhakar (Sudhakar et al., 2022)	2022	☆☆☆☆	☆☆	☆☆	8☆
Silver (Silver et al., 2020)	2020	☆☆☆☆	☆	☆☆	7☆
Traube (Traube, Silver, Gerber, et al., 2017)	2017	☆☆☆☆	☆☆	☆☆	8☆
Andrew (Koth et al., 2022)	2022	☆☆☆	☆☆	☆☆	7☆
Dervan (Dervan et al., 2020)	2019	☆☆☆	☆	☆☆	6☆
Patel (Patel et al., 2017)	2017	☆☆☆☆	☆	☆☆	7☆
Traube (Traube, Silver, Reeder, et al., 2017)	2017	☆☆☆	☆	☆☆	6☆
Traube (Traube, Ariagno, et al., 2017)	2017	☆☆☆☆	☆	☆☆	7☆
Christian (Christian et al., 2022)	2022	☆☆☆	☆	☆☆	6☆
Siegel (Siegel et al., 2021)	2021	☆☆☆☆	☆	☆	6☆
Alvarez (Alvarez et al., 2018)	2018	☆☆☆☆	☆	☆	6☆
Huang (Huang et al., 2023)	2023	☆☆☆	☆☆	☆☆	7☆
Yontem (Yontem et al., 2021)	2021	☆☆☆☆	☆	☆☆	7☆
Dervan (Dervan et al., 2022)	2022	☆☆☆	☆	☆☆	6☆

*Note:* Study quality was appraised using the Newcastle–Ottawa Scale across three domains: selection, comparability, and outcome. Each star (☆) represents one point awarded for meeting specific methodological criteria. The total score ranges from 0 to 9, with 7–9 stars indicating high quality, 5–6 stars indicating moderate quality, and ≤ 4 stars indicating low quality. All studies included in this review scored between 6 and 8, representing moderate to high methodological quality.

### 3.4. Predictive Ability of Delirium

#### 3.4.1. Mechanical Ventilation

**FIGURE 2:**
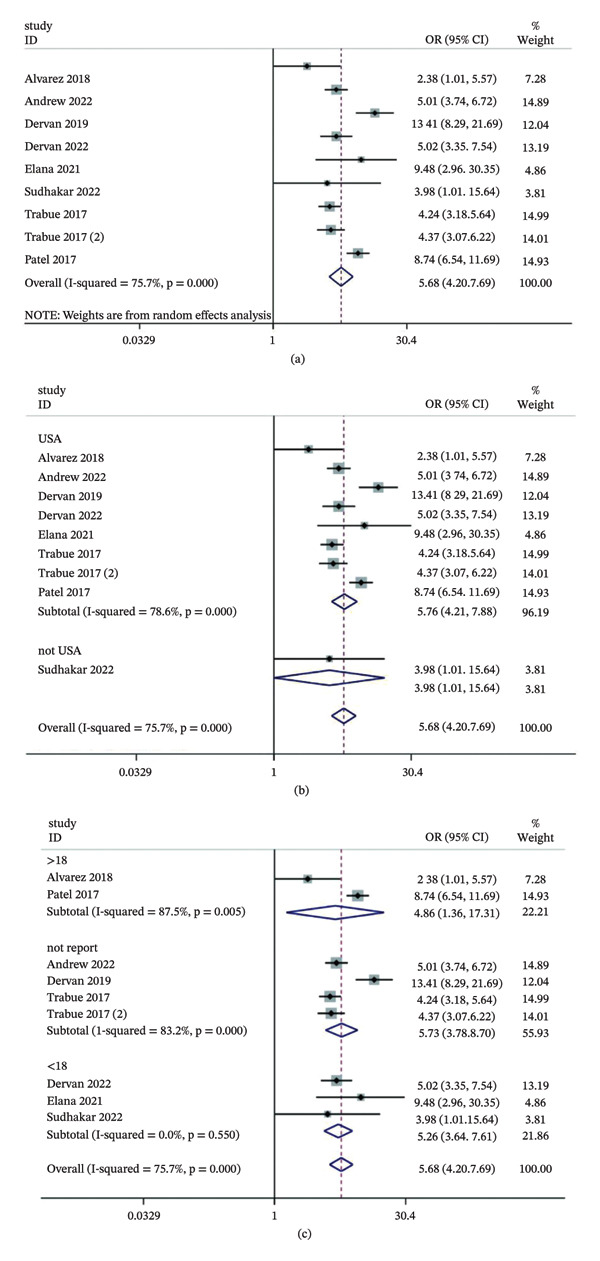
Association between delirium and mechanical ventilation risk in pediatric ICU patients. (a) Overall forest plot of the pooled odds ratio (OR); (b) subgroup analysis by study location; (c) subgroup analysis by patient age. Notes: The diamonds represent the pooled effect size, and the horizontal lines indicate 95% confidence intervals (CIs). Abbreviations: OR, odds ratio; CI, confidence interval; ICU, intensive care unit.

**FIGURE 3:**
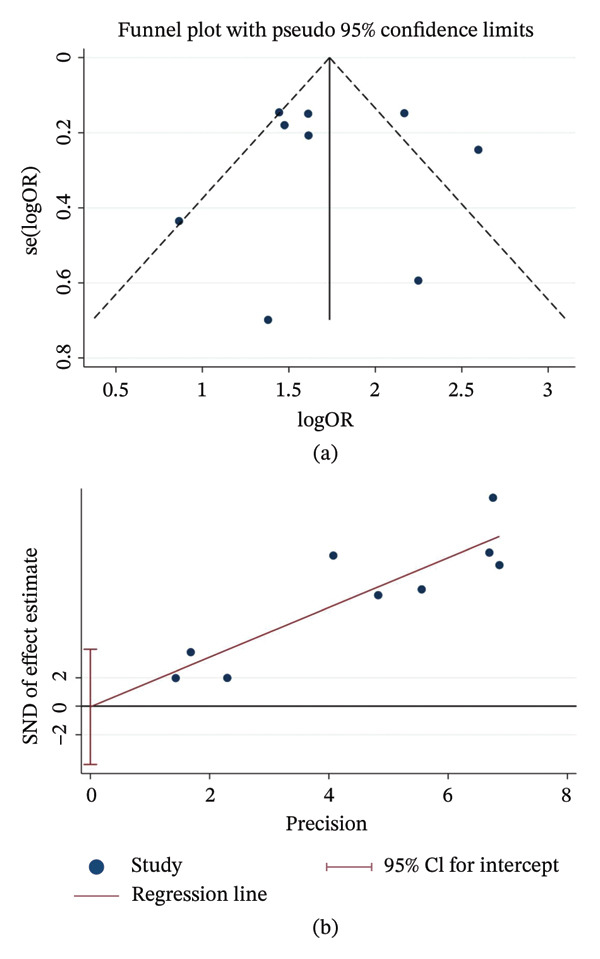
Publication bias assessment for mechanical ventilation risk. (a) Funnel plot; (b) Egger’s linear regression test. Notes: The symmetrical distribution and the *p* value from Egger’s test indicate the absence of significant publication bias. Abbreviations: OR, odds ratio; SE, standard error.

Nine articles examined the ability of delirium to predict the risk of mechanical ventilation. The pooled findings showed that children experiencing delirium in the ICU were roughly 5.7 times more likely to require mechanical ventilation (OR = 5.68, 95% CI: 4.20–7.69, *p* < 0.01), albeit with substantial between‐study heterogeneity (*I* ^2^ = 75.7%, *p* < 0.01) (Figure 2a). Subgroup analyses indicated that both study region and patient age significantly influenced the pooled effect sizes (Figure 2b–c). Funnel plots and Egger’s test (*p* = 0.977) revealed no evidence of publication bias (Figure 3a–b).

#### 3.4.2. ICU Length of Stay

**FIGURE 4:**
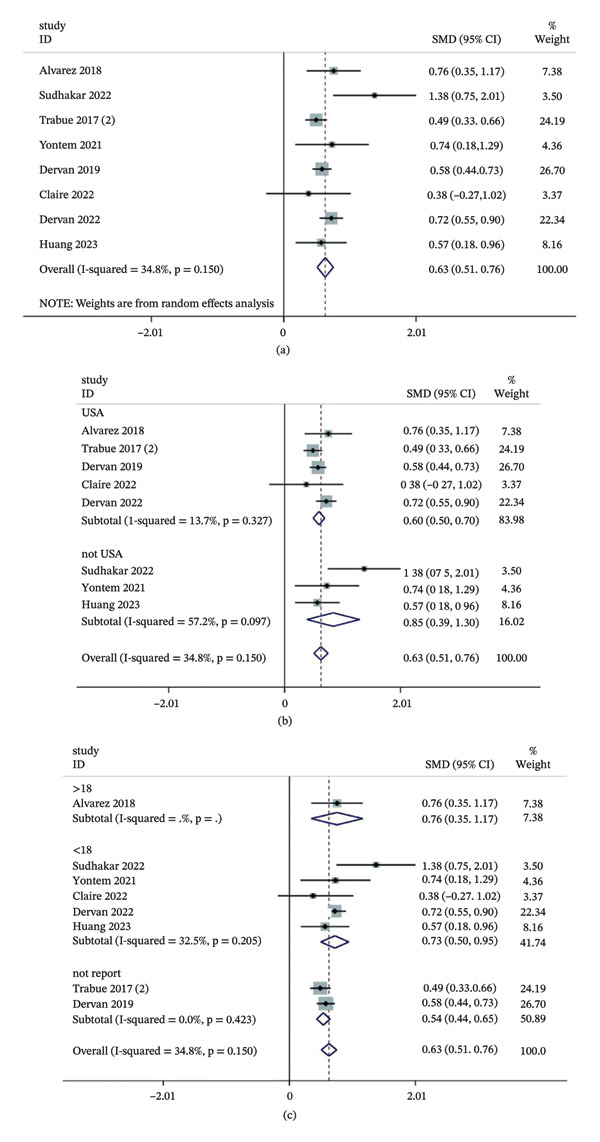
Association between delirium and ICU length of stay in pediatric. (a) Overall forest plot of the pooled odds ratio (OR); (b) subgroup analysis by study location; (c) subgroup analysis by patient age. Notes: The diamonds represent the pooled effect size, and the horizontal lines indicate 95% confidence intervals (CIs). Abbreviations: OR, odds ratio; CI, confidence interval; ICU, intensive care unit.

**FIGURE 5:**
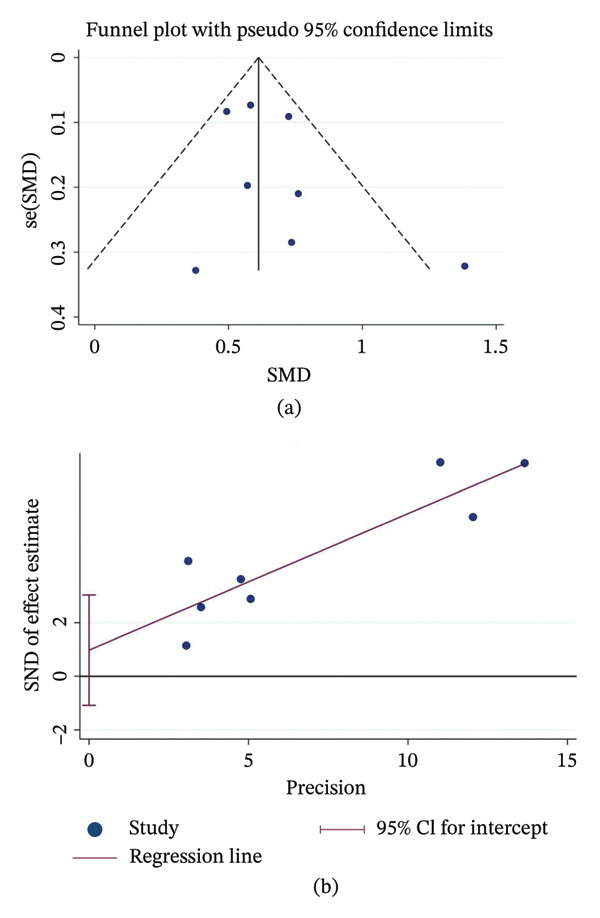
Publication bias assessment for ICU length of stay in pediatric. (a) Funnel plot; (b) Egger’s linear regression test. Notes: The symmetrical distribution and the *p* value from Egger’s test indicate the absence of significant publication bias. Abbreviations: OR, odds ratio; SE, standard error.

Eight studies investigated the impact of delirium on this outcome. The pooled results indicated that delirium prolonged ICU stay by approximately 0.6 days in children (SMD = 0.63, 95% CI: 0.51–0.76, *p* < 0.01) with modest heterogeneity (*I* ^2^ = 34.8%, *p* = 0.150) (Figure 4a). Subgroup analyses showed that both study region and patient age significantly influenced the pooled effect sizes (Figure 4b–c). Funnel plots and Egger’s test (*p* = 0.29) revealed no evidence of substantial publication bias (Figures 5a–b).

#### 3.4.3. Duration of Mechanical Ventilation

**FIGURE 6:**
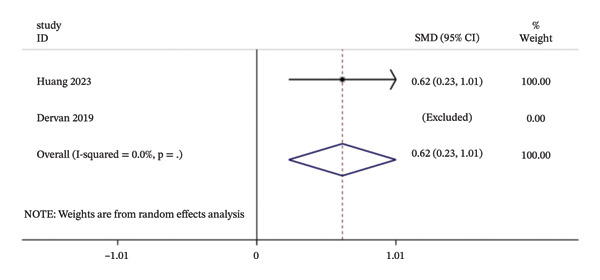
Forest plot for the duration of mechanical ventilation. Notes: This plot displays the available data from individual studies. Due to limited data and high clinical heterogeneity, a descriptive synthesis was prioritized in the results. Abbreviations: SMD: standardized mean difference; CI: confidence interval.

Data on the link between duration of mechanical ventilation and delirium were available from only two studies; however, the results were not meaningful because one study was excluded. Figure 6 illustrated the meta‐analysis results.

#### 3.4.4. Mortality

**FIGURE 7:**
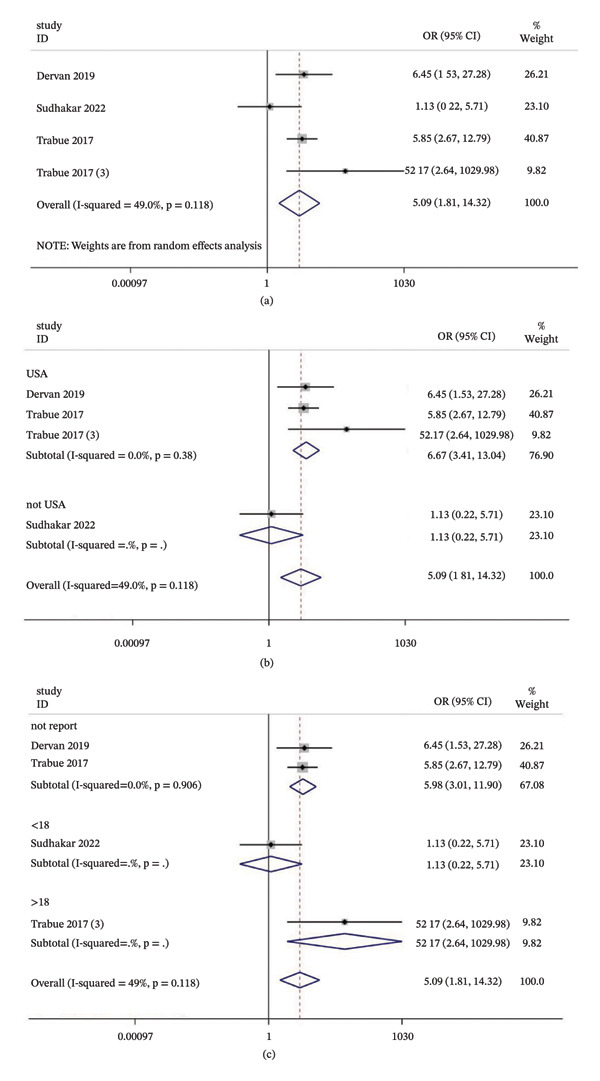
Association between delirium and mortality risk. (a) Overall forest plot of the pooled odds ratio (OR); (b) subgroup analysis by study location; (c) subgroup analysis by patient age. Notes: The diamonds represent the pooled effect size, and the horizontal lines indicate 95% confidence intervals (CIs). Abbreviations: OR, odds ratio; CI, confidence interval; ICU, intensive care unit.

**FIGURE 8:**
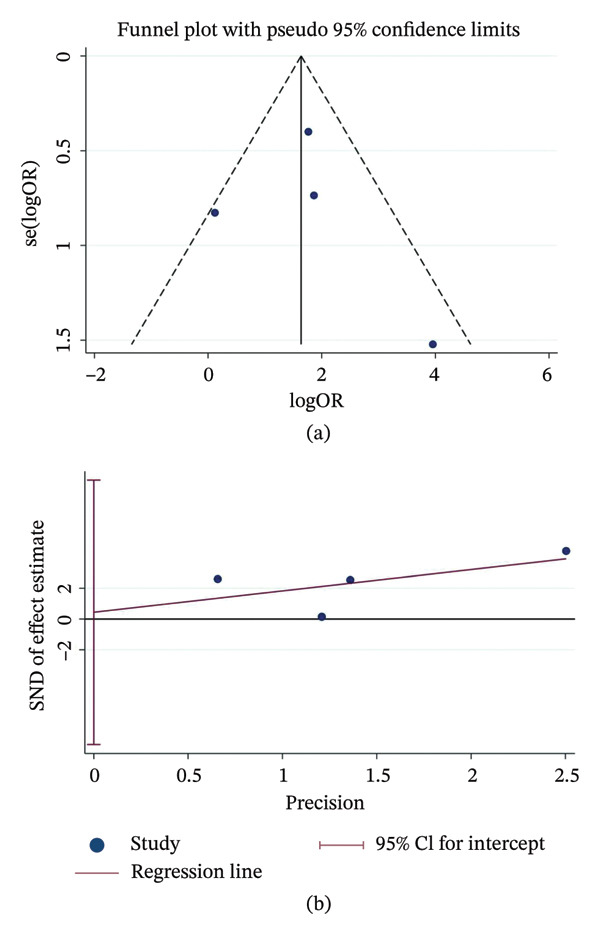
Publication bias assessment for mortality risk. (a) Funnel plot; (b) Egger’s linear regression test. Notes: The symmetrical distribution and the *p* value from Egger’s test indicate the absence of significant publication bias. Abbreviations: OR, odds ratio; SE, standard error.

Four studies explored the link between delirium and mortality. The pooled estimate indicated that children with delirium faced roughly a five‐fold higher risk of ICU death (OR = 5.09, 95% CI: 1.81–14.32, *p* < 0.01) with moderate heterogeneity (*I* ^2^ = 49.0%, *p* = 0.118) (Figure 7a). Subgroup analyses showed that both study region and patient age significantly influenced the pooled mortality effect (Figure 7b–c). Funnel plots and Egger’s test (*p* = 0.847) revealed no evidence of publication bias (Figure 8a–b).

#### 3.4.5. Quality of Life After Discharge

The ability of delirium to predict quality of life after discharge was examined in three articles. Because these three articles used different scales to assess quality of life, a meta‐analysis could not be performed, and we carried out a descriptive analysis. Silver et al. [26] used the Infant‐Toddler Quality of Life (IT‐QOL) questionnaire to assess postdischarge quality of life. The results showed that children with delirium scored lower than those without delirium at both 1 and 3 months after discharge. Huang et al. [35] applied the Chinese version of the Pediatric Quality of Life Inventory (PedsQL) 3.0 Cardiac Module and found that children without delirium had better scores than those with delirium at discharge and three months after surgery. However, by six months postsurgery, no significant differences between the two groups were observed. Dervan et al. [37] assessed children with severe functional or developmental disabilities using the Functional Status II‐R (FS II‐R), while other children were evaluated with the PedsQL 4.0 Generic Core Scales or the PedsQL Infant Scales. Their findings indicated that delirium was linked to a reduced quality of life in children assessed through PedsQL, but this association was not seen in children evaluated with FS II‐R. This discrepancy might result from the inherently higher baseline risk of quality of life decline in children with severe disabilities or from other clinical factors.

## 4. Discussion

This meta‐analysis presents strong evidence that delirium in children admitted to the ICU is closely associated with poor clinical outcomes, such as an elevated likelihood of requiring mechanical ventilation, increased mortality, and longer stays in ICUs. The pooled ORs indicate that children with delirium are over 5 times more likely to require mechanical ventilation (OR = 5.68, 95% CI: 4.20–7.69) and face a fivefold higher risk of death (OR = 5.09, 95% CI: 1.81–14.32). Furthermore, children with delirium tend to stay in the ICU approximately 0.61 days longer. A systematic review by Salluh et al. [4] found that patients aged over 16 years diagnosed with delirium had significantly greater in‐hospital mortality, prolonged mechanical ventilation, and longer ICU admissions, findings that are consistent with the results observed in children. Additionally, Goldberg et al. [38] found that adult patients with delirium had a strong association with long‐term cognitive impairment and increased healthcare costs, supporting the broader evidence that delirium serves as a key predictor of both disease severity and adverse long‐term outcomes.

From a clinical perspective, these data suggest that delirium is more than a transient neurobehavioral disturbance; it is a marker of critical illness in children. The occurrence of delirium is indicative of underlying systemic deterioration and can serve as an early warning sign of clinical worsening [28]. Timely identification through proven screening methods such as the CAPD, combined with multidisciplinary management—including environmental modifications such as sleep promotion and noise reduction, family involvement, and individualized nursing strategies—has been shown to effectively reduce the adverse outcomes associated with delirium [27, 32].

However, the geographical concentration of the included studies—predominantly from North America (78.6%, 11/14)—warrants a nuanced interpretation of our findings across different medical environments. Our subgroup analyses indicated that the study region significantly influenced the pooled effect sizes, which may stem from variations in regional nursing models and resource availability. For instance, the emphasis on multidisciplinary collaboration [11] and the implementation of standardized nursing protocols [12] are hallmark strategies in many North American PICUs, potentially leading to higher detection rates and more structured interventions. Conversely, in regions where such resources are constrained, the “family involvement” strategy highlighted in our review [10, 32] may play an even more critical role as a low‐cost, high‐impact nonpharmacological intervention. Furthermore, the significant barriers to delirium management identified in existing literature—such as low compliance with screening protocols [39, 40]—may manifest differently depending on local nursing workloads and institutional support. Therefore, while our results provide a robust framework, clinical application should be adapted to local healthcare infrastructures, prioritizing either resource‐intensive protocols or environment‐based, family‐centered strategies [9, 32] as appropriate.

Furthermore, pediatric‐focused research suggests that delirium adversely affects long‐term quality of life. Children who develop delirium frequently experience cognitive, behavioral, and functional deficits following discharge [26, 28, 35]. Children who experience delirium often exhibit cognitive, behavioral, and functional deficits after discharge, underscoring the importance of early detection and intervention. Although current data are limited and heterogeneous due to variations in assessment tools, emerging evidence indicates that preventing and managing delirium could significantly improve long‐term outcomes.

From a nursing management standpoint, these results underscore the critical need to integrate systematic delirium screening into routine ICU care. Implementing routine assessments with validated tools and providing targeted training for healthcare providers can enhance early recognition and management of delirium, ultimately reducing the risk of adverse outcomes. Developing standardized protocols and leveraging technological advances, such as real‐time monitoring, can further support timely interventions [33].

In summary, our analysis substantiates the critical impact of delirium on pediatric ICU outcomes and reinforces the imperative for proactive screening, early intervention, and multidisciplinary care to improve the prognosis and quality of life of affected children.

## 5. Limitations and Future Outlook

### 5.1. Limitations

This study has several limitations. First, all included studies were observational cohort designs. While this design is ethically necessary and methodologically appropriate for investigating delirium as a naturally occurring exposure (since RCTs would be infeasible and unethical), observational studies are inherently susceptible to residual confounding. Therefore, the observed associations between delirium and adverse outcomes should be interpreted as predictive rather than causal. Second, the overrepresentation of studies from North America (78.6%, 11/14) may limit the generalizability of our findings to other regions with different healthcare systems, cultural contexts, and patient populations. Disparities in nursing staffing, sedation practices, and the feasibility of family‐centered interventions across regions could influence both the occurrence of delirium and the efficacy of nursing management. Third, the subgroup analyses by study location and patient age were exploratory and not preregistered in the INPLASY protocol; these analyses were performed to explore sources of heterogeneity after data collection rather than being prespecified. Consequently, the findings from these subgroup analyses should be interpreted with caution and are hypothesis‐generating rather than confirmatory. Future studies should prespecify subgroup hypotheses to avoid data‐driven bias. Fourth, none of the included studies reported outcomes stratified by delirium subtypes (e.g., hyperactive, hypoactive, and mixed) or severity; all treated delirium as a binary outcome (present/absent). This prevented us from exploring whether different delirium phenotypes are associated with distinct risks for mortality, mechanical ventilation, or ICU length of stay. Fifth, heterogeneity in delirium assessment tools (although most used CAPD) and outcome measures across studies may introduce bias and complicate interpretation of results. Sixth, the inclusion of some adolescent patients older than 18 years may introduce heterogeneity, although this reflects real‐world clinical settings in pediatric ICUs. These limitations highlight the need for more rigorous, standardized multicenter research with prespecified subgroup analyses, stratification by delirium phenotype, and consistent use of validated outcome measures.

### 5.2. Challenges and Opportunities

Despite the clear benefits of early delirium identification, significant challenges remain in pediatric ICU settings. A lack of professional training in delirium assessment and low compliance with screening protocols are major barriers to effective delirium management [39]. Alarmingly, one study found that only 2% of pediatric ICU patients were routinely screened for delirium [40]. Addressing these challenges requires institutional commitment to training programs, resource allocation, and the development of standardized screening protocols. By overcoming these barriers, healthcare teams can improve early detection and intervention, ultimately enhancing patient outcomes.

### 5.3. Nursing Practice and Leadership Implications

Nurses play a crucial role in preventing and managing delirium through regular screening, environmental modifications, and family‐centered care strategies. The consistent use of reliable assessment tools, such as the CAPD, is essential for accurate and timely diagnosis. Environmental adjustments—including noise reduction and sleep promotion—can significantly decrease the incidence of delirium. Moreover, family‐centered rehabilitation strategies empower caregivers to participate actively in their child’s recovery, which can improve both short‐term and long‐term outcomes. Embedding these practices into routine nursing workflows ensures a comprehensive and holistic approach to delirium care, ultimately enhancing patient safety and recovery.

Nursing leaders must advocate for the adoption of evidence‐based delirium care practices by securing institutional support and fostering interdisciplinary collaboration. This involves obtaining resources for staff training, developing standardized protocols, and cultivating a culture of continuous quality improvement. Leaders should promote data‐driven evaluation of delirium interventions to inform practice adjustments and drive systemic change, prioritizing patient safety and recovery. By empowering nurses and encouraging teamwork, nursing leadership can significantly elevate the quality of pediatric critical care and promote sustained improvements in patient outcomes.

### 5.4. Future Directions

Future research should prioritize large‐scale multicenter studies to clarify delirium outcomes in children and validate intervention strategies. Longitudinal studies exploring delirium’s long‐term impact on cognition, emotion, and functional status are imperative to inform comprehensive rehabilitation. Importantly, future research should prioritize the prospective collection and reporting of delirium subtype and severity data. Multicenter studies designed with sufficient statistical power to perform subgroup analyses by delirium phenotype are urgently needed. Such data would enable a more granular understanding of outcome differences associated with different delirium types and facilitate the development of targeted, phenotype‐specific nursing interventions. Evaluating family‐centered and technology‐enhanced care models could optimize management. Addressing these gaps will enable nursing teams to develop evidence‐based protocols and deliver optimal care for critically ill children.

## 6. Conclusion

This systematic review and meta‐analysis demonstrates that delirium significantly affects nursing‐related outcomes in children admitted to the ICU, including a substantial increase in the likelihood of requiring mechanical ventilation, mortality, and prolonged ICU stays. While current evidence associates delirium with longer durations of mechanical ventilation and diminished quality of life after discharge, further research is needed to confirm these relationships. These findings underscore the critical importance of early detection and comprehensive nursing care to mitigate the adverse effects of delirium and support patient recovery and well‐being.

Nurses play a pivotal role by performing routine screenings, optimizing the care environment, and implementing family‐centered rehabilitation programs. Institutions should integrate delirium management protocols into nursing workflows to enhance early identification and treatment, alongside promoting standardized quality‐of‐life assessments to ensure holistic care for pediatric ICU patients. Through strengthening interdisciplinary collaboration and prioritizing delirium care, healthcare teams can significantly improve recovery trajectories and long‐term outcomes for critically ill children.

## Author Contributions

Cheng Yang: conceptualization, methodology, software, investigation, writing–original draft, and writing–review and editing. Qin Zeng: supervision, software, project administration, and writing–review and editing. Jiawei Li: data curation, formal analysis, visualization, and writing–review and editing.

## Funding

The work was supported by grant from the Sichuan Science and Technology Program [grant number 2022YFS0268].

## Disclosure

The authors confirm strict adherence to the Journal’s statistical guidelines. As a condition of submission, the authors assume full responsibility for ensuring the appropriateness of selected statistical methods, their correct implementation, and accurate interpretation. The funders had no role in study design, data collection and analysis, decision to publish, or preparation of the manuscript.

## Conflicts of Interest

The authors declare no conflicts of interest.

## Supporting Information

Additional supporting information can be found online in the Supporting Information section.

## Supporting information


**Supporting Information** This Supporting Information includes one file: Supporting Information 1, which presents the detailed search strategies used for each database (PubMed, Embase, CINAHL, and Cochrane Library). No supporting figures or tables are included in this submission.

## Data Availability

The data that support the findings of this study are available from the corresponding author upon reasonable request.
