# Supplementary material for: Impact of Delirium on Pediatric Critical Care Outcomes and Management Strategies: A Systematic Review and Meta‐Analysis
Source: J Nurs Manag. 2026 May 25;2026:9915593. doi: 10.1155/jonm/9915593 (PMC13201867; doi:10.1155/jonm/9915593)
Supplement: Supplementary file 1 — Supporting Information This Supporting Information includes one file: Supporting Information 1, which presents the detailed search strategies used for each database (PubMed, Embase, CINAHL, and Cochrane Library). No supporting figures or tables are included in this submission. [file JONM-2026-9915593-s001.docx]

# Supplementary Material. Search strategy used in database search

| **Database** | **Step** | **Query** | **Results** |
| --- | --- | --- | --- |
| PubMed | #1 | **"Delirium"[Mesh] OR "Delirium" OR "Subacute Delirium" OR "Delirium, Subacute" OR "Deliriums, Subacute" OR "Subacute Deliriums" OR "Delirium of Mixed Origin" OR "Mixed Origin Delirium" OR "Mixed Origin Deliriums"** | 22,132 |
|  | #2 | **"Child"[Mesh] OR "Adolescent"[Mesh] OR "Pediatrics"[Mesh] OR Child, Preschool OR paediatr* OR pediatr* OR infant* OR toddler* OR Teen OR Teenager OR Youth** | 4,182,083 |
|  | #3 | **"Cohort Studies"[Mesh] or Cohort Studies or Cohort Study or Studies, Cohort or Study, Cohort or Concurrent Studies or Studies, Concurrent or Concurrent Study or Study, Concurrent** | 2,494,893 |
|  | #4 | **(("Delirium"[Mesh] OR "Delirium" OR "Subacute Delirium" OR "Delirium, Subacute" OR "Deliriums, Subacute" OR "Subacute Deliriums" OR "Delirium of Mixed Origin" OR "Mixed Origin Delirium" OR "Mixed Origin Deliriums") AND ("Child"[Mesh] OR "Adolescent"[Mesh] OR "Pediatrics"[Mesh] OR Child, Preschool OR paediatr* OR pediatr* OR infant* OR toddler* OR Teen OR Teenager OR Youth)) AND ("Cohort Studies"[Mesh] OR Cohort Studies OR Cohort Study OR Studies, Cohort OR Study, Cohort OR Concurrent Studies OR Studies, Concurrent OR Concurrent Study OR Study, Concurrent)** | 4,931 |
| Embase | #**1** | **'delirium'/exp** | **41,327** |
|  | #**2** | **'delirium':ab,ti OR 'subacute delirium':ab,ti OR 'delirium, subacute':ab,ti OR 'deliriums, subacute':ab,ti OR 'subacute deliriums':ab,ti OR 'delirium of mixed origin':ab,ti OR 'mixed origin delirium':ab,ti OR 'mixed origin deliriums':ab,ti** | **29,521** |
|  | #**3** | **'child'/exp OR 'adolescent'/exp OR 'pediatric'/exp** | **4,138,627** |
|  | #**4** | **'child, preschool':ab,ti OR 'paediatr*':ab,ti OR 'pediatr*':ab,ti OR 'infant*':ab,ti OR 'toddler*':ab,ti OR 'teen':ab,ti OR 'teenager':ab,ti OR 'youth':ab,ti** | **1,184,728** |
|  | #**5** | **'cohort analysis'/exp OR 'cohort analysis'** | **1,096,317** |
|  | #**6** | **'cohort studies':ab,ti OR 'cohort study':ab,ti OR 'studies, cohort':ab,ti OR 'study, cohort':ab,ti OR 'concurrent studies':ab,ti OR 'studies, concurrent':ab,ti OR 'concurrent study':ab,ti OR 'study, concurrent':ab,ti** | **518,962** |
|  | #7 | **#1 OR #2** | **49,761** |
|  | #8 | **#3 OR #4** | **4,527,481** |
|  | #9 | **#5 OR #6** | **1,189,427** |
|  | #10 | **#7 AND #8 AND #9** | **295** |
| CINAHL | S1 | **(MM "Delirium") OR ( "Delirium" OR "Subacute Delirium" OR "Delirium, Subacute" OR "Deliriums, Subacute" OR "Subacute Deliriums" OR "Delirium of Mixed Origin" OR "Mixed Origin Delirium" OR "Mixed Origin Deliriums" )** | **49,823** |
|  | S2 | **( (MM "Child+") OR (MM "Adolescence+") OR (MM "Hospitals, Pediatric") ) OR ( Child, Preschool OR paediatr* OR pediatr* OR infant* OR toddler* OR Teen OR Teenager OR Youth )** | **3,185,732** |
|  | S3 | **(MM "Prospective Studies+") OR ( Prospective Studies or Cohort Studies or Cohort Study or Follow Up Study or Follow-Up Studies or Incidence Studies or Incidence Study or Longitudinal Studies or Longitudinal Study or Prospective Research or Prospective Study )** | **3,365,824** |
|  | S4 | **S1 AND S2 AND S3** | **289** |
| Cochrane Library | #1 | MeSH descriptor: [Delirium] explode all trees | 1,423 |
|  | #2 | (Delirium or Delirium of Mixed Origin or Mixed Origin Delirium or Mixed Origin Deliriums or Delirium, Subacute or Subacute Deliriums or Subacute Delirium or Deliriums, Subacute):ti,ab,kw | 5,682 |
|  | #3 | MeSH descriptor: [Child] explode all trees | 77,623 |
|  | #4 | MeSH descriptor: [Adolescent] explode all trees | 118,643 |
|  | #5 | MeSH descriptor: [Intensive Care Units, Pediatric] explode all trees | 1,519 |
|  | #6 | (Child or Children paediatr* or infant* or pediatr* or toddler* or Teen):ti,ab,kw | 198,643 |
|  | #7 | MeSH descriptor: [Cohort Studies] explode all trees | 186,471 |
|  | #8 | (Cohort Study or Study, Cohort or Concurrent Studies or Study, Concurrent or Concurrent Study or Studies, Concurrent or Cohort Study or Studies, Cohort or Incidence Studies or Incidence Study or Studies, Incidence or Study, Incidence or Study, Historical Cohort or Historical Cohort Study or Studies, Historical Cohort or Historical Cohort Studies or Cohort Study, Historical or Cohort Studies, Historical or Analysis, Cohort or Cohort Analyses or Cohort Analysis or Analyses, Cohort):ti,ab,kw | 187,431 |
|  | #9 | #1 or #2 | 5,682 |
|  | #10 | #3 or #4 or #5 or #6 | 276,436 |
|  | #11 | #7 or #8 | 337,482 |
|  | #12 | #9 and #10 and #11 | 397 |
